# Recent Advances in Health Benefits of Bioactive Compounds from Food Wastes and By-Products: Biochemical Aspects

**DOI:** 10.3390/ijms24032019

**Published:** 2023-01-19

**Authors:** Valeria Sorrenti, Ilaria Burò, Valeria Consoli, Luca Vanella

**Affiliations:** 1 Department of Drug and Health Science, University of Catania, 95125 Catania, Italy; 2 CERNUT-Research Centre on Nutraceuticals and Health Products, University of Catania, 95125 Catania, Italy

**Keywords:** food wastes, health, bioactive compounds, circular economy, natural extracts

## Abstract

Bioactive compounds, including terpenoids, polyphenols, alkaloids and other nitrogen-containing constituents, exert various beneficial effects arising from their antioxidant and anti-inflammatory properties. These compounds can be found in vegetables, fruits, grains, spices and their derived foods and beverages such as tea, olive oil, fruit juices, wine, chocolate and beer. Agricultural production and the food supply chain are major sources of food wastes, which can become resources, as they are rich in bioactive compounds. The aim of this review is to highlight recent articles demonstrating the numerous potential uses of products and by-products of the agro-food supply chain, which can have various applications.

## 1. Introduction

In recent years, in functional foods, plant-derived nutraceuticals and dietary supplements have embodied health-promoting ingredients able to enhance human well-being. Functional foods, dietary supplements and nutraceuticals contain, beyond the nutritional components, bioactive compounds with health benefits [1,2,3,4,5]. Phenolic compounds (PCs), including terpenoids (carotenoids and phytosterols), polyphenols, alkaloids and other nitrogen-containing constituents (glucosinolates), represent the majority of so-called bioactive compounds and they can be found in vegetables, fruits, grains, spices and their derived foods and beverages such as tea, olive oil, fruit juices, wine, chocolate and beer [6]. The largest group of non-nutrient dietary PCs are secondary metabolites synthesized by plants under normal and stress conditions [7]. PCs include polyphenols that, according to their structural characteristics, are subdivided into three major classes: flavonoids, non-flavonoids and phenolic acids [8].

Polyphenol compounds exert various beneficial effects arising from their antioxidant and anti-inflammatory properties [9]. The compounds behave as chain breakers of lipid peroxidation reactions and metal (Fe^2+^) chelators, which inhibit oxidant enzymes (such as xanthine oxidase) and modulate cellular signaling processes that interfere with protein and lipid kinase pathways [10]. Additionally, polyphenols allow for the restoration of the endogenous antioxidant pool (superoxide dismutase, glutathione peroxidase and catalase), which is reduced due to a high rate of elimination [11]. To maintain redox homeostasis, adequate amounts of antioxidants need to be supplied by the diet or dietary supplements [12]. Based on several epidemiological studies, a diet rich in antioxidant polyphenol compounds (mainly flavonoids, phenolic acids, lignans, stilbenes, tannins and anthocyanins) or the intake of polyphenol-rich derivatives, seems to favor a delay in the onset of degenerative diseases including cardiovascular injuries, cancers, metabolic disorders and brain dysfunctions, in which oxidative stress plays a key role [12,13]. Several studies report that dietary intake of foods (apple, onion, orange and grapefruit) rich in flavanones (naringenin) and flavonols (quercetin) resulted in a decreased risk of cardiovascular damage and tumor proliferation [14,15,16,17,18,19]. In particular, in recent years, several active systems have been developed using the flavonoid quercetin, exploiting mainly its antioxidant capacity to prevent oxidation phenomena in food products [20]. Besides its established antioxidant activity, some authors report the antimicrobial activity of this flavonoid against Gram-positive and Gram-negative bacteria, in vitro and by means of a shelf-life study on food products [21,22]. Additionally, the anti-inflammatory activity of flavonoids, especially quercetin glycoside compounds, was investigated. Montone et al. [23] reported a significant reduction in cytokine production during infection of the human macrophage cell line U93, especially IL8, when quercetin is more exposed on the surface of nanoparticles. Indeed, these components play a crucial role in the prevention of non-communicable diseases, such as cardiovascular pathologies, type 2 diabetes, some types of cancer and neurodegenerative diseases [24]. The intake of phenolic compounds is, hence, part of a healthy diet. Nowadays, due to the knowledge of positive bioactive compounds’ impact on health, many people around the world observe a vegetarian diet as a lifestyle and, consequently, they consume more vegetables and agricultural products. However, agricultural production and the food supply chain are major sources of waste biomass: the huge amount of agro-industry wastes/by-products, including peels, seeds, leaves, stems, shells and skins, is generated during all the stages of the food life cycle, from the cultivation, through industrial manufacturing to market [25].

For this reason, it has been estimated that wastes from vegetables and fruits are destined to increase in the near future, leading to an inevitable environmental burden. Therefore, proper disposal of wastes is necessary to avoid undesirable and dangerous outcomes for the environment [26]. However, since proper disposal of wastes represents a cost, the circular economy, a model of economy where by-products are not waste but resources to be valorized and reused, ref. [27] has proposed as a solution to minimize raw material input and waste production. Due to their peculiar composition, agri-food processing residues are recognized as materials of high biorefinery potential, offering a range of opportunities for the sustainable production of food, feed, chemicals and energy [28].

The aim of this review is to highlight recent articles demonstrating numerous potential uses of products and by-products of the agro-food supply chain, which can have various applications such as in animal feed production and the preparation of compost, and as certain bio-fuels and bioethanol [29,30]. Recently, extracts derived from vegetable and fruit wastes have become an interesting topic for researchers as they represent a way to exploit waste production for the development of new formulations, which can exert significant beneficial effects on human health [31,32,33,34,35]. Compared to non-processed waste, phenolic content in waste extracts increases significantly during processing, particularly when air drying is used to remove water [36]. However, since the co-extraction of phenolic compounds with compounds that may be toxic, such as emerging pollutants (EPs), could occur, the use of green methodologies leading to phenolic-rich extracts with low environmental impact should be preferred [37,38].

In this review, we report conventional and green extraction methods and procedures and discuss the advantages and drawbacks of their use; we focus on the recovery of products with high added value from olive trees, citrus fruits, tomato, pomegranate, avocado, mango and hazelnut, which represent some of the most popular vegetables or fruits eaten worldwide.

## 2. Extraction Methods

**Figure 1:**
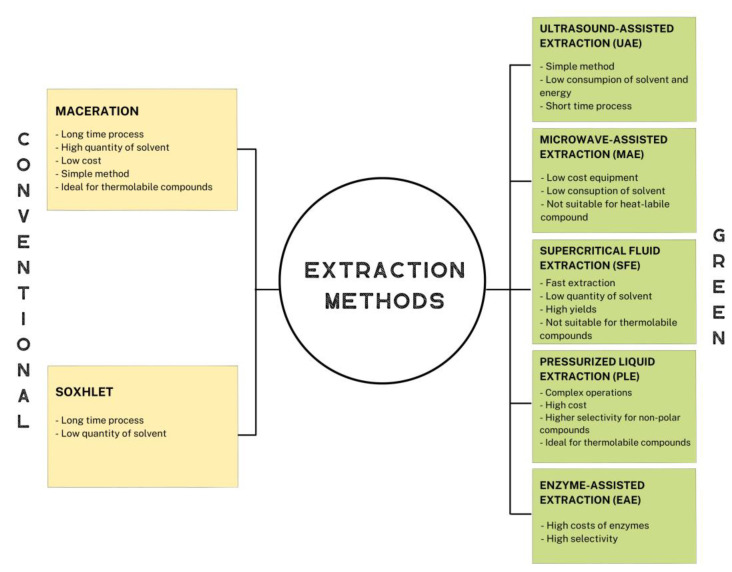
Advantages and limitations of conventional and green extraction methods.

In a circular economy context, it is important to mention the application of environmentally friendly and ecological extraction methods. Extraction techniques used for the recovery of bioactive compounds can be distinguished into two categories: conventional methods and green extraction methods (Figure 1). In any case, all extractive techniques have the following objectives: (a) to extract the desired target compounds, minimizing their degradation, and without altering their properties; (b) to obtain the extract in the most stable and pure form; (d) to increase the extraction yield of the desired compounds; and (e) to reduce costs and process time [39].

### 2.1. Conventional Extraction Method

To obtain bioactive compounds from fruits or from vegetables, a wide range of conventional methods of extraction are available nowadays such as percolation, maceration, decoction, Soxhlet extraction and hydro-distillation. The main conventional techniques used to recover bioactive compounds from waste matrixes include maceration and Soxhlet extraction.

#### 2.1.1. Percolation

Percolation is the procedure most frequently used to extract active ingredients in the preparation of tinctures and fluid extracts. It is a continuous process in which the saturated solvent is constantly being replaced by fresh solvent [40].

#### 2.1.2. Maceration

Maceration has found use in the extraction of bioactive compounds from waste due to its advantage of being applicable to thermolabile compounds [40]. It consists of mixing the solid matrix with the appropriate extraction solvent. First, the solid material is reduced to small pieces in order to increase the contact surface area with the solvent. The system can be placed under agitation or a heat source to increase the extraction yield [41]. Maceration is a simple method but the duration of extraction time is long and the quantity of solvent required is more.

#### 2.1.3. Decoction

Decoction is a water-based preparation used to extract active compounds from medicinal plant sections. A liquid preparation is made by boiling the plant material with water. Decoction is the method of choice if working with tough and fibrous plants, barks and roots and with plants that contain water-soluble chemicals. Decoction cannot be used for the extraction of thermolabile or volatile components [42].

#### 2.1.4. Soxhlet Extraction

The technique of Soxhlet extraction was originally developed by Fraiz Ritter Von Soxhlet for the extraction of lipids from a solid material, and since then, it has also been adapted for bioactive compounds from various natural sources. Soxlet extraction consists of placing the solid material in the central part of the extractor, which is directly connected to a distillation flask containing the solvent. The latter is brought to the boil by passing it through the extraction chamber and into the condenser. Falling back as a condensate, the solvent is charged with solute and drawn out through a side duct. The solution is then heated until the solvent is enhanced [43,44]. Compared to maceration, this technique requires a smaller quantity of solvent because it undergoes a recycling process. It is a very simple method which does not require filtration after extraction. However, the process time is very long [45].

#### 2.1.5. Hydro-Distillation

Hydro-distillation is a traditional method for the extraction of plant-derived compounds that does not exploit organic solvents in the process. In hydro-distillation, plant materials are packed in a still compartment and water is added in a sufficient amount, and then, brought to boil. This process involves three main physico-chemical processes: hydro-diffusion, hydrolysis and decomposition by heat. However, one of the main limitation of this technique is that it cannot be used for the extraction of thermolabile compounds [40].

### 2.2. Eco-Friendly Green Extraction Method

The sustainable development of green methods is based not only on the idea of turning waste to wealth but also on the concept of “green chemistry”, whose goal is to reduce or eliminate the use and generation of hazardous substances [46].

Conventional extraction methods are still the main approach for obtaining bioactive compounds, but this technology is very often accompanied by high expenditure and the disposal of energy and toxic chemicals. The eco-friendly separation of bio-compounds from agro-industrial waste, supported by the use of an eco-sustainable and low-cost solvent such as water, is obviously attractive from both socio-environmental and economic points of view [47].

Certainly, the term green denotes a class of extraction methods that have several advantages over traditional methods. These include the use of small quantities of solvents, especially green solvents, and more ecological techniques. Green solvents are obtained from renewable sources and are biodegradable, water being among them [48] as it is a solvent that is safe for health, inexpensive and widely available. In addition, the penetration of water through the sample matrix can be improved, thus increasing mass transfer kinetics by raising the temperature and pressure [49]. Ethanol is another suitable solvent that is cheap and renewable and is, in fact, produced via the fermentation of biological material. Its use is ideal for the extraction of phenolic compounds and it has great selectivity for the extraction of oils [50]. New types of solvents such as deep eutectic solvents (DESs) have been developed in recent years. A DES is a mix between a halide salt or another hydrogen bond acceptor (HBA), and a hydrogen bond donor (HBD). They have shown great potential for the extraction of bioactive compounds/molecules from fruit and vegetable waste such as peels [51]. They can represent an alternative to conventional organic solvents such as benzene, toluene, xylene and methanol due their greater efficiency, safety and green extraction [52,53].

#### 2.2.1. Ultrasound-Assisted Extraction (UAE)

Ultrasound-assisted extraction (UAE) is based on the working principle of acoustic or ultrasonic cavitation. The sound waves disperse into the solvent containing the matrix to be extracted, collapsing into the surface of the solids and causing their breakage. It is a method that consumes low amounts of solvent, low energy, and has a low extraction time. On the other hand, it may not be suitable for thermolabile compounds because heat is generated during the process [54,55].

#### 2.2.2. Microwave-Assisted Extraction (MAE)

Microwave-assisted extraction (MAE) is a method that uses non-ionizing electromagnetic waves in a frequency range from 300 MHz to 300 GHz. The advantage of this technique is the use of low-cost equipment that requires reduced extraction time and solvent quantity. The microwaves heat the sample/solvent mixture, generating pressure within the solid particle, resulting in breakage and facilitating solvent penetration and extraction of the analyte [54,55,56].

#### 2.2.3. Supercritical Fluid Extraction (SFE)

In this case, the solvent used is in the super-critical state, a condition in which the fluid lies between the gaseous and liquid state. For this purpose, temperature and pressure parameters are controlled. One of the most commonly used solvents in this method is carbon dioxide (CO_2_). This technique has high costs and complex operations but, on the other hand, has high selectivity for non-polar compounds and is ideal for thermolabile compounds [55,57,58].

#### 2.2.4. Pressurized Liquid Extraction (PLE)

Pressurized liquid extraction (PLE) consists of the use of pressurized solvents at high temperatures. High pressure allows for the use of small quantities of solvent, rapid extraction and high yields. High temperatures could damage thermolabile compounds [43].

#### 2.2.5. Enzyme-Assisted Extraction (EAE)

Cellulase, xylanase and pectinase are examples of the enzymes most used for the extraction of bioactive compounds. These enzymes degrade the cell wall structure, releasing the bioactive molecules. This method has high selectivity; however, the enzymes are not only expensive, but also complicated to handle, and strict control of pH and temperature is required to achieve the optimal enzyme yield [55,59].

## 3. Bioactive Compounds Obtained from Wastes and Innovative Applications

Table 1 shows the main bioactive compounds present in the most popular vegetables or fruits eaten worldwide, which are discussed in the following paragraph.

### 3.1. Olea europaea L.: Bioactive Compounds Found in By-Products, Waste and Leaf, and Beneficial Effects

*Olea europaea* L. is a fruit tree cultivated in the entire Mediterranean region. The main product of *Olea europaea* L. fruits is extra-virgin olive oil, which is known for its nutritional properties and healthy effects, especially against cardiovascular diseases due to the presence of bioactive compounds [60]. Additionally, olive by-products and leaf are an extraordinary source of bioactive compounds [61] that can be recovered by exploiting green technologies and reused for food, agronomic, nutraceutical and biomedical applications [27]. In this context, a study by Talhaoui et al., 2015 demonstrated that olive leaves contain a wide variety of phenolic compounds such as oleuropein, hydroxytyrosol, tyrosol, cumaric acid, ferulic acid, caffeic acid, vanillic acid, rutin, verbascoside, luteolin, quercetin, dimethyloleuropein and ligstroside [62]. The main component of all the constituents of olive leaf extract is oleuropein. Its content varies from 17 to 23% [63] and it has antimicrobial, antioxidative and anti-inflammatory effects, as well as the ability to treat oxidative stress and inflammatory-related diseases such as cardiovascular disease, hepatic disorder, obesity and diabetes [64,65,66,67,68,69]. This phenol can inhibit low-density lipoprotein oxidation and lipoxygenases [70]. Furthermore, as reported by different studies, olive leaf extract is known for its ability to improve lipid metabolism to ameliorate obesity-related conditions [70,71,72].

Moreover, Acquaviva et al. showed the antiproliferative effect of oleuropein in prostate cell lines, suggesting its possible use as an adjuvant agent in the treatment of prostatitis, in order to prevent the transformation of hypertrophic to cancerous cells [73].

In a recent study by Cecchi et al., it was shown that the phenolic fraction extracted from olive into olive oil never exceeds 2%, remaining almost completely in the milling by-products [74,75].

Among the wastes from oil production, in addition to olive mill wastewaters, there is patè, a particular dried olive pomace containing high amounts of oleuropein and hydroxytyrosol. In detail, oleuropein and hydroxytyrosol are valuable compounds for their high antioxidant capacity and for their metal-chelating and free-radical-scavenging activities. The high antioxidant activity of oleuropein and hydroxytyrosol is due to their ability to scavenge reactive oxygen species (ROS) and stabilize oxygen radicals with an intramolecular hydrogen bond [76]. During fruit maturation and olive oil production, the enzymatic conversion of oleuropein, catalyzed by β-Glucosidase, leads to the formation of its bioactive derivative, hydroxytyrosol [77]. Due to its hydrophilic character, hydroxytyrosol is abundant in olive oil by-products and, in particular, in olive oil wastewaters, thus representing a precious source from which to extract this valuable compound [78]. Additionally, hydroxytyrosol from olive leaves was shown to be a powerful human monoamine oxidase (hMAO) inhibitor. MAO enzymes are useful targets for both depression and neurodegeneration treatments, they catalyze the oxidative deamination of endogenous and dietary amines and are involved in dopamine metabolism. Thus, hydroxytyrosol and other bioactive compounds able to inhibit MAOs have been suggested as potential molecules for the treatment of Alzheimer’s, Parkinson’s and other neurological diseases [79,80,81].

Studies that report eco-friendly extraction methods for olive leaves are very interesting. A study conducted by Benincasa et al. proved that from the sole use of water, it was possible to obtain extracts of molecules of great health and pharmacological value. They used an aqueous extraction procedure to retrieve bioactive compounds from olive leaves, such as oleuropein, hydroxytyrosol, tyrosol, verbascoside, lutein and rutin, by using ultrapure, microfiltered and osmosis-treated water. In this context, it possible to open up new opportunities for ecological approaches designed for bioeconomy and circular economy models [47].

In our previous study, the solid/liquid extraction of olive leaves on adsorbent polymeric resins, followed by elution using hydro-alcoholic solutions and the recovery of alcohol (ethanol), allow us to obtain concentrated aqueous extracts that were spray-dried to obtain the powdered standardized extracts. The olive leaf extract (OLE) obtained was rich in bioactive compounds such as oleuropein and polyphenols, it showed good antioxidant capacity, it was able to reduce the accumulation of free fatty acids and could act as cholesterol-lowering agent. Therefore, OLE obtained from the waste and by-products of agricultural production could represent a solution not only by emphasizing these sources but also by giving to the olive industry new possibilities that allow them to increase the added value of their production [82].

### 3.2. Citrus Fruit Waste: Beneficial Health Effects and Its Utilization

Citrus fruits, such as oranges, grapefruits, lemons, limes, tangerines and mandarins, are among the most popular fruits cultivated across the globe [83]. The *Citrus* genus belongs to the *Rutaceae* family, subfamily *Aurantioideae*, and it as been known for its beneficial effects on health for centuries. These plant groups contain many beneficial nutrients and bioactive compounds, which could reduce the risk of various chronic diseases (e.g., cardiovascular diseases and metabolic disorders) [84,85,86]. Many studies have highlighted the nutritional and health-promoting properties of citrus fruits, showing several biological functions such as antimicrobial, anticancer, antidiabetic, antiplatelet aggregation and anti-inflammatory activities [87]. Moreover, recently reported by Eman et al. was the chemopreventive effect of orange peel extract against cyclophosphamide-induced organ toxicity in an in vivo model [88].

Citrus fruits are mostly cultivated in tropical and subtropical regions due to their soil and favorable climatic conditions that are suitable for their growth. Every year, the production of citrus fruits increases and, consequently, the waste resulting from their processing increases. About a fifth of the total *Citrus* cultivars are subjected to industrial processes, and only 45% of the total fruit weight is exploited, whereas the peel, pulp and seeds are disposed of [89]. Moreover, in order to improve the quality of the fruit, tree pruning is practiced, implying the production of large quantities of leaves, a residue that increases the already large amount of *Citrus* waste [90,91].

Citrus fruit waste includes seeds, peels, pomace, membrane residues, secondary juice (obtained by pressing the residual pulp after the primary juice extraction) and leaves. It has important economic value; in fact, several alternatives utilizations have been proposed for managing citrus fruit waste. All these wastes can be used as sources of bioactive compounds, for food preservation and as renewable source of energy employed in the food, pharmaceutical and cosmetic industries [92,93,94].

The peel of *Citrus* contains up to 5000 mg/g of phenolic content, more than the edible portion of the fruit [95]. The major bioactive compounds present in citrus fruits belong to the subclasses terpenoids and phenolics. Carotenoids and limonoids are the main examples of the terpenoids, whereas flavonoids (naringenin, naringin, hesperidin, quercetin and rutin), phenolic acids and coumarins are the main examples of phenolic compounds present in citrus fruits and in their wastes [96,97].

Two classes of carotenoids can be distinguished: xanthophylls, which are oxygenated carotenoids such as lutein and violaxanthin, and carotenes, such as β-carotene and lycopene [98,99]. They are precursors of vitamin A, which is involved in epithelial tissue growth and strengthening of the immune system, and promotes the proper functioning of vision [100,101]. Hydroxybenzoic (gallic, vanillic and syringic acids) and hydroxycinnamic acids (caffeic, ferulic, p-coumaric and sinapic acids), classified as phenolic acids, are also present and they are known to possess high levels of free-radical-scavenging activity [102,103].

Among the Citrus fruits, we can also include the red orange cultivars, which are particularly rich in red pigments belonging to the anthocyanin class such as cyanidin-3-glucoside (C3G) [104].

As for other wastes, the residue from the industrial processing of red oranges, called *pastazzo* (peels, pulps and seeds), also has a higher content than Citrus fruit of flavanones, hydroxycinnamic acids and anthocyanins, especially C3G, and has numerous nutraceutical properties. In our previous study, we assayed the beneficial effects of a powdered standardized extract obtained via the solid/liquid extraction of peels, pulps and seeds of red oranges on adsorbent polymeric resins, followed by elution using hydro-alcoholic solutions and the recovery of alcohol (ethanol). The extraction ended with a spray-drying process that does not require the use of organic solvents [82]. Our extract, rich in bioactive compounds such as C3G, showed a good antioxidant capacity and was able to reduce lipid accumulation in FFA-exposed HepG2 cells. So, it can be useful in preventing and counteracting the complications of hepatic steatosis. The Citrus fruit plants contain essential oils, which are plant secondary metabolites and contain an assembly of volatile compounds, generally found in the oil sacs of citrus peels and cuticles [105]. Due to their antioxidant, antimicrobial and insecticidal properties, essential oils have been used as bio-preservatives in all types of food with the purpose of extending their shelf-life [106].

Moreover, aqueous orange peel extract was shown to have different food applications such as supplementation in minced beef to suppress lipid oxidation [107] and milk for increasing the antioxidant activity and total polyphenol content and decreasing the total microbial count [108]. Interestingly, strong inhibition of MAO activity was observed by citrus peels infusions, which could make them good dietary means for the prevention and management of neurodegenerative conditions [109,110].

Since they are considered generally-recognized-as-safe (GRAS) products by the US Food and Drug Administration (FDA), these essential oils can be suitable in sustainable green chemistry for replacing chemical additives [106]. There are more than 200 components identified in the lipid components of *Citrus*, which are commonly made up of aldehydes, ketones, esters, acids, terpenes and alcohols [111]. The antimicrobial ability of these essential oils and major components against bacteria and yeasts showed that these are more effective on Gram-positive bacteria, Gram-negative bacteria and yeasts [112]. The peel waste and seed oil of *Citrus* are also employed as natural resources for biofuel production such as bioethanol, biodiesel and biogas. Fermentation and anaerobic digestion are used for the bioconversion of citrus peels into biofuel. For instance, from mandarin peel was prepared bioethanol (50–600 L/1000 kg), utilizing pre-treatment with steam explosion and microbial fermentation [113]. Moreover, orange peel waste was used for the production of bioethanol via simultaneous pre-treatment with acid-catalyzed steam explosion and separate enzymatic hydrolysis plus fermentation with *Saccharomyces cerevisiae.* Likewise, it was realized that the developed seed oil coming from *Citrus reticulata* (mandarin) meets all the standards for the production of biodiesel [114]. A trans-esterification process was carried out from *Citrus reticulata* (mandarin) seed-derived oil using sodium methoxide with methanol; this process led to a biodiesel yield of about 76.93% [115]. The use of waste in biorefineries could provide: (a) significant economic benefits such as the recovery of both energy and products with high added value, the creation of new jobs in new businesses and significant savings on landfill costs, and (b) environmental benefits thanks to the reduction in greenhouse gases [116].

Citrus fruit wastes are extensively used in the cosmetic industry both for their content of bioactive compounds and of natural oils. Being a rich source of bioactive compounds, antioxidants, vitamins (vitamin C and E) and polyphenolic compounds, nowadays, agro-industrial waste is also utilized as an active ingredient to obtain skincare products [117]. The antioxidants present in citrus peel help in delaying skin aging and the aids in reducing oxidative damage, as well as skin-related issues such as acne, wrinkles, dark spots, etc. Zahra [118]. A study conducted by Wuttisin et al. confirmed that orange peel extract can be applied in the cosmetic industry to increase the value of orange peel waste. The research focused on checking the anti-tyrosinase activity of orange (*Citrus sinesis* L.) peel extract, with the purpose of formulating whitening cream that helps in averting the production and accumulation of melanin pigment. It was found that orange peel-based cream could reduce melanin pigment by 17.33% [119]. Citrus seeds, which are rich in natural oils, are also utilized in the cosmetic industry for the preparation of soaps, body lotions, body sprays and other cosmetic products [120]. Atolani et al. developed a soap containing citrus seed oil that exhibited remarkable anti-microbial, anti-fungal, anti-parasitic and antioxidant properties [84]. Moreover, the use of citrus seed oils to obtain cosmetic products might prevent exposure to synthetic chemicals. Among citrus wastes, citrus pomace can also be included as a source of pectin [121]. The high pectin content of citrus residues has been used in the field of nanotechnology as an encapsulating agent to improve the stability, as well as the efficiency, of encapsulation [122]. For instance, citrus peel waste-based oil emulsion was fabricated utilizing a hydro-soluble component of citrus waste (pectin), citric acid, Ca^2+^ ion and ascorbic acid. The study reported that the prepared emulsion was stable and had a reduced droplet size [123].

In addition, in vitro studies suggested high bio-accessibility of encapsulated resveratrol in contrast to free resveratrol. Additionally, enhancement in the antioxidant properties of resveratrol was observed [124]. Therefore, it can be suggested that the citrus by-products may be employed as encapsulants.

### 3.3. Avocado Fruit Wastes: Utilization of By-Products

Avocado (*Persea americana* Mill.) is a subtropical/tropical fruit native to Mexico and Central America, and is widely produced and consumed worldwide. The avocado fruit is a drupe constituting an epicarp (peel), mesocarp (pulp) and endocarp (seed), whose size, shape, color and phytochemical content depend on the genotype. It belongs to the *Lauraceae* family and the genus *Persea*, of which there are more than 150 known species [125].

As reported by the FAO, about six million tons of avocado are produced annually around the world [126]. Avocado wastes include peel, seed and defatted paste and they are considered a source of environmental contamination. However, as reported above, wastes can be exploited due to their wealth of protein, fibers and numerous bioactive compounds [127,128].

As reported by Yahia & Woolf, 2011, avocado pulp is a good source of protein, carbohydrates, dietary fibers, vitamins (such as vit. C, E and K, choline, niacin and pantothenic acid) and minerals [129]. However, the avocado fruit is best known for its high lipid content (about 12–24% of the whole fruit). The lipids most present are mainly monounsaturated fatty acids including oleic acid (62.14%), palmitic (17.2%), linoleic (11.11%) and palmitoleic (7.34%) and polar lipids, such as glycolipids and phospholipids, which are essential, especially for cell membranes. The peel and seed of the avocado, which are by-products, are rich sources of carbohydrates (seed: 42–81%; peel: 43–81%), lipids (seed: 3–15%; peel: 2–9%), proteins (seed: 0.14–9%; peel: 0.17–8%), fibers (seed: 2–4.2; peel: 1.3–55%), minerals (seed: 1.3–4.3%; peel: 1.5–6.0%) and various other bioactive compounds [125]. Avocado fruit is rich in phenolic compounds, which are particularly abundant in its peel and seed [130]. Phenolic acids, flavonoids and tannins are the most relevant types of phenolic compound present in avocado fruit [131,132]. The lipid fraction of avocado fruit and its by-products contains polyhydroxylated fatty alcohol derivates (PFAs), which are more polar than fatty acids due to their hydroxyl groups. Rosenblat et al. identified two main avocado-derived PFAs: 1-ace-toxy-2,4-dihydroxy-heptadec-16-ene (PFA-A) and 1-acetoxy-2,4-dihydroxy-heptadec-16-ene (PFA-B), with relative concentration percentages of 32% and 54% in the seeds, and 51% and 29% in the pulp, respectively [133]. Acetogenins, a type of PFA whose chemical structure contains a long-chain fatty acid with a terminal γ-lactone, are characteristically found in avocado mesocarp and seed. The acetogenin profile and concentration have been shown to vary in avocado seed during early ripening, with no change in the mesocarp [134].

Avocado fruit and its by-products contain several other minor components such as carotenoids, alkaloids, phytosterols and tocopherols among others. Thus, the current scientific evidence suggests that extracts from avocado residues (peel, seed coat and seed extracts) could be used as functional ingredients to be added to nutraceuticals or novel food products [135,136,137].

Nevertheless, regular consumption of foods rich in these types of phenolic compound has been associated with health-related benefits due to their antioxidant, antiproliferative and anti-inflammatory activities. Some studies have suggested that avocado residues might be used to obtain phenolic-rich extracts with antiproliferative properties, playing a potential role in cancer prevention/treatment. Regarding avocado peel extracts, it has been reported that they may induce apoptosis in MDA-MB-231 cells due to increased activation of caspase 3 and caspase 3 target protein PARP. On the other hand, avocado seed extracts have shown anti-inflammatory and antiproliferative activities against the HCT-116 (colorectal carcinoma) and the HepG-2 (liver cancer) cell lines in a dose-dependent manner [138]. Avocado fruit is rich in lipid molecules such as PFAs which have received attention as useful cytotoxic molecules; indeed, some studies have shown their cytotoxic effects on various cell types, which have been associated mainly with apoptosis induction. For example, Llor and co-workers reported that fatty acids (FA), such as oleic acid (C18:1) and linoleic acid (C18:2), induced apoptosis in the human colon adenocarcinoma cells HT-29 and Caco-2 [139]. Avocado’s anti-cancer activities have been associated with more than 20 groups of bioactive compounds, among them long-chain lipid molecules such as avocatins, pahuatins, persenins. Ethanolic extract of immature avocado fruit (with a higher proportion of persin) showed cytotoxic activity against different cell lines: lung carcinoma cells (A549), kidney cells (A498), pancreas (PaCa2), breast adenocarcinoma (MCF-7), colon (HT-29) and prostate (PC-3) [140]. Avocatin B, predominant in avocado seed and peel, had greater selectivity towards cancer cells [141]. A study by MónicaLara-Márquez et al. evaluated the anticancer activity of a lipid-rich extract (LEAS) from native Mexican avocado seed in the colon adenocarcinoma cell line Caco-2. Their results showed that LEAS expressed its cytotoxicity at a concentration of 28 μg/mL through apoptosis induction and the activation of caspase 8 and 9. The cytotoxic effect was also related to the loss of mitochondrial membrane potential, the inhibition of FA oxidation, and ROS production. Furthermore, LEAS showed the immunomodulatory activity in Caco-2 cells because the secretion of cytokines IL-6, IL-8 and IL-10 was stimulated while IL-1β secretion was inhibited [142].

Another study described the photoprotective and anti-inflammatory potential of PFA derived from the seed of avocado fruit. It evaluated the protective properties of PFA against UVB irradiation in cultured keratinocyte and in a human skin explant. The photo protective effect was shown through increased cell viability and a decreased number of sunburnt cells in human skin explants. Furthermore, enhanced DNA repair was demonstrated by measuring the removal of cyclobutane pyrimidine dimers (CPD), one of the most important characteristics of DNA damage and mutagenesis. The exposure of keratinocytes to 20 mJ/cm^2^ UVB induced the formation of CPD, as was measured immediately after irradiation. CPD removal for 24 h was obtained in the cells treated with PFA [133].

### 3.4. Health Effects of Extracts Obtained from Pomegranate Wastes

*Punica granatum* L. (pomegranate) is a fruit that grows in arid and semiarid zones. Popular zones for growing pomegranate trees are Iran, India and the Mediterranean countries such as Turkey, Egypt, Tunisia, Spain and Morocco. The only genus is *Punica* and the predominant species is called *P. granatum* [143]. The fruit is formed of peel and red pulp that surrounds the seeds. Pomegranate, in addition to as a fruit, is available in many other forms, such as juice, powdered capsules that are derived from seeds, dry tea or a drink made from leaves or seeds, peel, leaves and flowers. Only the pulp is edible as it is, but the remining solid waste also contains various bioactive and nutritional molecules [144]. For example, there are phytoestrogen compounds in pomegranate seeds that have sex steroid hormones such as tocopherol, testosterone, stigmasterol, b-estrolsitosterol and 17-a-estradiol, similar to those in humans [145]. Moreover, about a fifth of white seeds consist of oil and the most abundant fatty acids found in pomegranate seed oil are punicic acid, linoleic acid and oleic acids. Fernandes et al. reported that the largest portion of pomegranate seed oil is punicic acid (65%), which is a conjugated 18-carbon fatty acid with three double bonds. The remaining is linoleic acid, which makes up 7% of pomegranate seed oil, while other less abundant compounds are sterols, tocopherols and cerebrosides [146,147]. The peel and juice are rich in polyphenols. The largest classes include tannins and flavonoids, which have pharmacological potential due to their antioxidative activities. Ellagitannins are a class of hydrolyzable tannins that are present in fruits and nuts such as pomegranates, black raspberries, raspberries, strawberries, walnuts and almonds [148]. Punicalagin, punicalin and gallagic acid are the most important ellagitannins found in the peel and juice of pomegranate [149]. All these ellagitannins have in common the ability to be hydrolyzed to ellagic acid, resulting in a prolonged release of ellagic acid into the blood following the ingestion of the fruit [150]. Classes of pomegranate flavonoids include anthocyanins, flavan3-ols and flavonols. Anthocyanins cause the red color of the juice, which is not found in the peel. Pomegranate juice also contains other polyphenols, such as anthocyanins (cyanidin, delphinidin and pelargonidin glycosides) and flavonols (quercetin, kaempferol and luteolin glycosides) [150]. In all the phases of the fruit’s life cycle, for example, during agricultural production, industrial manufacturing, and processing, wastes of pomegranate fruit are produced. Researchers’ interest in identifying natural products obtained from dietary plants has increased in recent years, so it is possible to benefit from pomegranate by-products as they are a rich source of bioactive compounds [151].

Pomegranate wastes and by-product extracts can be used in the prevention and treatment of several types of cancer [152]. Ellagitannins present in pomegranate are metabolized by the gut flora into smaller phenolic compounds such as ellagic acid and urothyroline A and B, and can reach different target organs where their beneficial action is exerted. As reported by Matthew B. Rettig et al., pomegranate extract (PE) inhibits cancer prostate cell growth and induces apoptosis via a mechanism involving the inhibition of NF-κB activity [153]. Furthermore, it is important to note that the NF-κB inhibitory effect of PE is necessary to induce the maximum induction of pro-apoptotic proteins (Bax and Bak) and the downregulation of anti-apoptotic proteins (Bcl-xL and Bcl-2) [145,154]. Ellagic acid appears to be responsible for the antioxidant and anticarcinogenic effect, arresting human prostate cancer cell invasion [155,156,157,158,159]. Moreover, ellagic acid from pomegranate peel was reported to decrease MAO-B activity and increase Nrf2 in rats with Parkinson’s disease [160].

Pomegranate waste extracts have been the subject of other studies in order to determine their beneficial effects and potential use. In a study conducted by Raffaele et al., an in vitro model of intestinal inflammation was developed using Caco-2 cells exposed to lipopolysaccharide (LPS). The obtained results revealed the potential role of bioactive compounds contained in pomegranate wastes in the prevention of inflammatory bowel disease (IBD). Such activity may be due to two different mechanisms: pomegranate extract’s ability to activate HO-1, which may be able to buffer the harmful effects of oxidative stress and reduce damage, and its ability to induce TIGAR (TP53-inducible glycolysis and apoptosis regulator). Furthermore, the potential beneficial effects of PE derived from waste were supported by the results obtained in vivo. Data highlighted the ability of the extract to reduce intestinal inflammation, preserve the colon length and histological features and reduce IL-6 levels compared to the dextran sulfate sodium (DSS)-induced IBD model group [161].

Our previous study demonstrated that 3T3-L1 pre-adipocytes treatment with pomegranate peel extract resulted in a significant reduction in lipid accumulation during cellular differentiation into adipocytes. These data suggested that bioactive compounds present in the extract are able to suppress adipocyte differentiation. Moreover, the combination treatment of extract and probiotic *L. rhamnosus* GG ATCC 53103 strain (LGG) was more effective in reducing intracellular lipid accumulation. These results evidenced that probiotics and polyphenols contained in extract obtained from pomegranate wastes may affect adipogenesis in vitro, demonstrating that the synergistic properties of combining foods such as pomegranate and probiotics may provide health benefits [162].

### 3.5. Hazelnuts: Health Benefits of Hazelnut Waste Extracts

Hazelnuts are the most popular nuts consumed worldwide. *Corylus avellana* L. is the scientific name. They are widely cultivated in Turkey, Spain and Italy [163]. Tonda di Giffoni and Tonda gentile delle Langhe are two specialities cultivated in Italy that have been awarded the PGI (Protected Geographical Indication) mark [164,165]. Shells and skins are the by-products that have been given considerable attention as sources of antioxidants [166,167]. The shell has low commercial value and is only used as a heating source after combustion [168]. The skin of hazelnuts, which accounts for 2.5% of their weight, is removed and discarded during roasting to improve the kernel flavor, color and crunch. The review of Sansone et al. highlights the potential of hazelnut by-products as a source of natural antioxidant and bioactive compounds with human health-beneficial effects [169]. In recent years, researchers have been interested in studying the exploitation of different lignocellulosic biomasses for the production of alcohols, acids, polyols, furans and aromatic compounds or for pharmaceutical and nutraceutical applications [170]. Activated carbon from shells, due to their high lignin content, can be used as an adsorbent material for heavy metals originating from the environment, such as lead [171,172], chromium, cadmium, zinc [173], nickel, [174] and copper [175]. Other studies have focused on the hemicellulosic portion of hazelnut shell (HS) extract, which contains xylans, precursors of xylooligosaccharides and arabino-xylooligosaccharides (AXOS) [176]. These compounds have been defined as prebiotics, i.e., non-digestible, non-vitalizing food-derived components that confer health benefits to the host associated with modulation of the microbiota [177]. These prebiotics promote the selective growth of bifidobacteria, lactobacilli and eubacteria in the intestinal tract and inhibit the growth of potentially harmful bacteria such as clostridia and enterobacteria [178]. It is also possible to exploit hazelnut shells for their high content of phenolic compounds. In this regard, a study conducted by Lelli et al. analyzed the composition of two hazelnut varieties, both in terms of total phenolic compound content and antioxidant capacity and in terms of metabolomics, in order to add economic value to hazelnut industry waste. The two varieties in question are the Tonda di Giffoni and the Tonda Gentile Romana. With regard to total polyphenol content and antioxidant capacity, no significant differences were found between the two varieties. Conversely, metabolomics data showed that the two varieties have different metabolic profiles. The biosynthetic pathways of flavonoids and phenylpropanones of the Tonda Romana were higher than in the Giffoni varieties. Instead, myricetin and syringetin compounds were more representative in the Giffoni cultivars [179]. Another study by Ottaggio et al. reports the presence of taxanes in a methanolic extract of hazelnut shells and leaves. Among these, paclitaxel, 10-deacetylbaccatin III, baccatin III, paclitaxel C and 7-epipaclitaxel were identified and quantified. Such chemotherapeutic agents demonstrated the ability of the extract to inhibit metaphase–anaphase transactivation in a human lung cancer cell line (SK-Mes-1) [180]. Another by-product of industrial hazelnut processing is roasted hazelnut husk, which is rich in phenolic compounds and especially in proanthocyanidins A and B. Its extract and fraction, enriched in proanthocyanidins, have been characterized and tested by Piccinelli et al. The high content of total phenolics (TP) (1.27 and 5.11 μmol GAE/mg, respectively) and proanthocyanidins (PA) (5.4 and 19.6 μmol CE/mg, respectively), gives the extract potent antioxidant activity with EC50 = 58.5 and 7.8 μg/mL, respectively, evaluated via a DPPH assay. In addition, the antifungal property of the extract was tested against Candida albicans after 48 h of incubation. The results show that, at a concentration of 3.0 μg/mL (corresponding to MIC2, i.e., the lowest concentration that produces 50% growth) and 5.0 μg/mL (corresponding to MIC0, the lowest concentration that does not produce growth), the extract reduced the number of fungal cells, but also inhibited the germination and generation of true hyphae compared with the control [169,181].

### 3.6. Tomatoes: Health Benefits of Tomato Waste Extracts

Tomato (*Solanum lycopersicum*) is one of the most popular vegetables worldwide with a high content of bioactive compounds. Tomato consumption is linked to health benefits that help in preventing some oxidative stress-related diseases such as cancer or cardiovascular diseases [182]. Tomato is consumed as a fresh food or processed in the food industry; therefore, the high consumption of tomatoes leads to a high accumulation of by-products such as tomato peels, seeds and small amounts of pulp that are removed during treatments. Tomato by-products contain polyphenols, tocopherols and carotenoids (mostly lycopene, β-carotene and lutein). Szabo et al. evaluated the phenolic compound and carotenoid content of ten tomato varieties’ processing wastes and determined the antioxidant and antimicrobial capacities of the obtained extracts [183]. Tomato peel extracts contained bioactive compounds such as lycopene, β-carotene, lutein and different phenolic compounds, and presented moderate antimicrobial activity. The authors concluded that tomato processing wastes could represent a low-priced antimicrobial agent for application in food packaging and storage. The carotenoids that occur in tomatoes are mainly lycopene and β-carotene. Previously, protocols for lycopene extraction from tomato wastes used traditional solvent extraction methods based on the use of different combinations of conventional organic solvent mixtures, which are recognized as a cause of environmental issues [98,184,185]. Subsequently, to reduce their use and adopt a green chemistry approach, more eco-compatible extraction processes operating under mild conditions, such as UAE, SFE and EAE, have been utilized [186,187].

Paulino et al., as an alternative application of tomato wastes, obtained a flour from tomato industrial by-product, dried using a conventional method which allowed them to reach a high content of nutraceutical compounds. The authors also used eco-friendly and food-grade solvents such as ethyl acetate to obtain extracts rich in phenolic compounds or in carotenoids. According to the authors, thanks to their metabolite content and antioxidant capacity, the flours could be added into new food products or the extracts could be used as a food additive [188].

Kumar et al. [189] reported that seed wastes generated during tomato fruit processing contain significant amounts of bioactive compounds that exhibit health-promoting activities. In vitro and in vivo studies with animal models demonstrated the antiplatelet, antioxidant, anticancer, antimutagenic, antimicrobial and neuroprotective effects of tomato seed extracts [190,191,192].

### 3.7. Mango Fruits: Health Benefits of Mango Waste Extracts

Mango is a tropical fruit belonging to the *Anacardiaceae* family. The mango-producing countries are mainly tropical and subtropical, including India, China, Thailand, Indonesia, the Philippines, Pakistan and Mexico.

*Mangifera indica* is the scientific name of the plant. Three parts can be distinguished in the fruit: a large seed (or endocarp) surrounded by a yellow-orange pulp that makes up about 40–65% of the average weight, and the peel (or epicarp). The edible part of the fruit is the pulp, the remaining part is usually discarded [193]. Mango pulp is the main consumable part of the mango and it is the source of nutritional compounds such as reducing sugars, amino acids, aromatic compounds and functional compounds, such as vitamins, pectin, polyphenols and anthocyanins [194]. Recently, it has been reported that mango intake exerts beneficial effects in slowing the progression and reducing the severity of IBD [195].

Mango processing generates peels and kernels as wastes, which also contain functional compounds. Mango peels contain protocatechuic acids, mangiferin and β-carotene, known for their antimicrobial, anti-diabetic, anti-inflammatory and anti-carcinogenic properties [193,196,197,198].

Mango peels have a high content of polyphenols, enzymes and vitamins C and E. In particular, ethylgallate and penta-*O*-galloyl-glucose are two of the bioactive compounds that have been isolated in the peel. In addition to having important antioxidant activity (hydroxyl radical (OH^•^), superoxide anion (^•^O_2_^−^) and singlet oxygen (^1^O_2_)-scavenging activities), gallate derivatives (e.g., penta-*O*-galloyl-glucoside) have been the subject of several studies that have demonstrated their bioactivity, including anticancer [199,200], antioxidant [201], anti-cardiovascular [202] and hepatoprotective effects [203].

In this regard, Kim et al. tested the antioxidant and cytoprotective properties of a mango peel extract on H_2_O_2_-induced oxidative damage in a human hepatocarcinoma cell line (HepG2) [204]. Mango peel extract exhibited significant antiproliferative effect against the tested cancer cell lines in a dose-dependent manner.

The mango kernel has higher antioxidant and polyphenolic amounts than the pulp and peel. It is used for oil extraction, and in combination with corn and wheat flour, it is employed in preparing nutraceuticals [205].

Ribeiro da Silva et al. demonstrated that Mango by-products showed higher levels of beta-carotene and lycopene, as well as anthocyanins and yellow flavonoids, when compared to the fruit pulps, confirming that agro-industrial by-products are sources of bioactive compounds [206]. Recently, Lauricella et al. demonstrated that the anti-cancer effect of *Mangifera indica* L. Peel extract (MPE) is associated with γH2AX-mediated apoptosis in colon cancer cells [207]. The authors characterized the polyphenolic profile of MPE and demonstrated, for the first time, the presence of lepidimoic acid, a pectic disaccharide, in mango peel. The results obtained by Lauricella et al. provided a new insight into the potential anti-tumor benefits of mango peel as a supportive strategy for antineoplastic therapies.

### 3.8. Grapevine: Health Benefits of Winemaking Waste Extracts

Winemaking is a multistage process entailing certain critical points which lead to the generation of several by-products and wastes. Among the organic wastes produced are the grape pomace, lees and stalk, which are released during the crushing phases, and wastewaters, which are produced following the pressing, filtration and stabilization processes. Considering its importance in the wine industry, grapevine (*Vitis vinifera* L.) is considered one of the most important fruit crops in the world. About 2000 different cultivars of *V. vinifera* are currently used for grape production all over the world [208,209].

The European leaders in wine production, including Italy, France and Spain, can produce up to a million tons of vinification solid residues and tens of millions of cubic meters of wastewater per year, the management of which has an important impact both from an environmental and economic perspective. Indeed, inadequate waste disposal can affect living beings, and water and soil quality, due to antinutritional substances, oxygen depletion and greater resistance to microorganisms [210].

Several studies report attempts that have been made globally to profit from such wastes by reusing them as soil fertilizers, distilled beverages, building construction materials, energy sources and livestock feed. Moreover, the huge amounts of bioactive compounds that can be found in winery waste have attracted the attention of experts from the pharmaceutical, cosmetic and food sectors [211,212].

Phenolic substances in *V. vinifera* may be divided into phenolic molecules (hydroxycinnamic and hydroxybenzoic acids) and polyphenolic compounds (flavonoids and stilbenes). Flavonoids contained in *V. vinifera*, such as anthocyanins, catechins, and flavonols, have been widely reported to possess homeostatic and astringent properties that are useful for the treatment of different conditions such as hemorrhoids, diarrhea, bleeding, circulatory diseases and varicose veins [213,214].

Bud-preparations, also referred to as bud-derivatives, are a recent type of botanical preparation derived from plant material and can be produced via meristematic plant tissues maceration in a mixed solution of solvents (usually ethanol, glycerol and water). Several health-promoting compounds have been found in different species of *Vitis vinifera* and its bud-derivatives such as alkaloids, amino acids, anthraquinones, coumarins, enzymes, glycosides, phenolic compounds and terpenes [214,215].

In the vision of sustainable production and profitable waste management, the use of grapevine pruning wood could be an opportunity to turn a by-product into an input for the bud-derivate production chain. Moreover, exploiting eco-sustainable and innovative extraction techniques combined with the traditional ones may positively influence the herbal companies and the single producers [216].

In a study by Donno et al., bud-derivatives produced using ultrasounds have been presented as excellent sources of phenolic compounds to be used both in the cosmetic and food industries. Their results showed very good recovery of polyphenols from ultrasound bud-extracts with a profile similar to the phytochemical pattern of the bud-derivatives obtained via traditional cold maceration. Moreover, the ultrasonic extraction was quite advantageous as it made it possible to reduce the extraction times from 21 days to 20 min [213,217].

Hübner and colleagues evaluated the activity of one extract (grape pomace extract, GPE) and two fractions (one chloroform, GPE-CHF; one ethyl acetate, GPE-EAF) obtained from by-products of the winemaking process of red grapes (*Vitis vinifera* L. cv. C. Sauvignon). GPE-EAF containing syringic acid and quercetin was able to elevate the sample’s action against UVB radiation by improving the SPF in vitro. Thus, it was concluded that winemaking waste could be considered a valid source of dermocosmetic ingredients for sunscreen products [218].

Due to the high content of phenols and polyphenols, the antioxidant activity of winemaking wastes has been widely explored; however, Maia et al. have also reported that ‘Pinot noir’ leaves have high nutritional potential both for human and animal consumption in addition to their potential exploitation as a source of bioactive compounds. Indeed, grapevine leaves were observed to contain a high content of fatty acids, with alpha-linolenic acid being the most abundant, and polyphenols such as caffeic acid, catechin, kaempferol, quercetin and resveratrol-derived flavonoids [219].

Additionally, kaempferol present in *V. vinifera* was also shown to be a selective inhibitor of MAO-A isoform, suggesting a potential use in neurological conditions [220]. Interestingly, *V. vinifera* pruning waste resveratrol-rich extract activity was evaluated in malignant and healthy cells, showing preferential selectivity through the induction of different cell death phenotypes. The tested extracts had a poly-pharmacological effect, which suggested a potential use for them in preventing malignancies such as cancer [221]. Pinot noir pomace extract was also observed to possess antioxidant, anti-inflammatory and antiproliferative effects both in vitro and in vivo [216].

Table 2 summarizes the main health benefits of the fruit wastes discussed in the present review.

## 4. Conclusions

The aim of the present review is to highlight results obtained in recent research that demonstrate the potential use of fruit and vegetable wastes as sources of bioactive molecules useful in preventing or treating chronic diseases, maintaining well-being or enhancing human health. Numerous scientific studies demonstrate the positive correlation between food and health. Furthermore, food wastes can become resources, as they are rich in bioactive compounds. The proposed review highlights the additional value of plant secondary metabolites, mainly as potential nutraceuticals for human health, and their ability to counteract several oxidative stress-associated diseases. Therefore, the agro-food industry produces large amounts of by-products that may possess added-value compounds. Consumers’ demand for healthier food and health products has increased in recent years, representing a challenge for the food and pharmaceutical industries. Additionally, the use of green techniques allows for the production of chemical-free compounds, which are recognized as safe and are preferred by consumers. However, the use of bioactive compounds is not limited to the formulation of health-related products, indeed they may have other applications such as in the cosmetic industry (i.e., the prevention and treatment of skin-related issues) [222] and in the food industry in order to develop functional food [223], to increase shelf-life [224,225] or to improve the quality of frying oil [226].

Vegetable wastes such as tomato or olive tree wastes, as well as fruit wastes such as citrus, pomegranate, avocado, mango and hazelnut wastes, being abundant and low-cost renewable resources, could be used to develop new nutraceutical and/or pharmaceutical products, and may have a positive economic and environmental impact.

## Figures and Tables

**Table 1:** Bioactive compounds present in most popular vegetables or fruits eaten worldwide.

Fruit	Bioactive Compounds
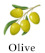 **Olive**	Oleuropein, hydroxytyrosol, tyrosol, cumaric acid, ferulic acid, caffeic acid, vanillic acid, rutin, verbascoside, luteolin, quercetin, dimethyloleuropein and ligstroside
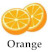 **Orange**	Carotenoids, limonoids, flavonoids (naringenin, naringin, hesperidin, quercetin, and rutin), hydroxycinnamic acids and anthocyanins, hydroxybenzoic (gallic, vanillic and syringic acids) and hydroxycinnamic acids (caffeic, ferulic, p-coumaric and sinapic acids) and coumarins
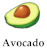 **Avocado**	Phenolic acids, flavonoids, tannins, carotenoids, alkaloids, phytosterols and tocopherols
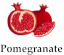 **Pomegranate**	Punicalagin, punicalin and gallagic acid anthocyanins (cyanidin, delphinidin and pelargonidin glycosides), flavonols (quercetin and kaempferol) and luteolin glycosides
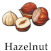 **Hazelnut**	Myricetin and syringetin, and proanthocyanidins A and B
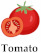 **Tomato**	Polyphenols, tocopherols, carotenoids (mostly lycopene, β-carotene and lutein)
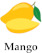 **Mango**	Ethylgallate and penta-*O*-galloyl-glucose lepidimoic acid
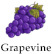 **Grapevine**	Hydroxycinnamic and hydroxybenzoic acids, anthraquinones, coumarins, alpha-linolenic acid, caffeic acid, catechin, kaempferol, quercetin, resveratrol and resveratrol-derived flavonoids

**Table 2:** Health benefits of fruit wastes.

Fruit	Wastes	Health Benefit	References
***Olea europaea***	Olive mill wastewaters, dried olive pomace, leaves	Anticancer, antimicrobial, antioxidative and anti-inflammatory effects.	[27,47,60,61,62,63,64,65,66,67,68,69,70,71,72,73,74,75,76,77,78,79,80,81,82]
***Citrus***	Seeds, peels, pulps, pomace, membrane residues, secondary juice, leaves	Antimicrobial, anticancer, antidiabetic, antiplatelet aggregation, anti-inflammatory, antifungal, antiparasitic and anti-oxidant activities. Able to reduce lipid accumulation.	[83,84,85,86,87,88,89,90,91,92,93,94,95,96,97,98,99,100,101,102,103,104,105,106,107,108,109,110,111,112,113,114,115,116,117,118,119,120,121,122,123,124]
***Avocado***	Peels, seeds, defatted paste	Anticancer, anti-inflammatory and antiproliferative activities.	[125,126,127,128,129,130,131,132,133,134,135,136,137,138,139,140,141,142]
***Pomegranate***	Peels, seeds	Anticancer, antioxidative and anti-inflammatory effects. Able to reduce lipid accumulation.	[143,144,145,146,147,148,149,150,151,152,153,154,155,156,157,158,159,160,161,162]
***Hazelnut***	Shells, skins	Antioxidant, anticancer and antifungal effects.	[163,164,165,166,167,168,169,170,171,172,173,174,175,176,177,178,179,180,181]
***Tomato***	Skins, seeds, pomace	Antiplatelet, antioxidant, anticancer, antimutagenic, antimicrobial and neuroprotective effects.	[98,182,183,184,185,186,187,188,189,190,191,192]
***Mango***	Peels, kernel	Antimicrobial, anti-diabetic, anti-inflammatory and anti-carcinogenic properties.	[193,194,195,196,197,198,199,200,201,202,203,204,205,206,207]
***Grapevine***	Peels, bud-derivatives, pomace, leaves	Antioxidant, homeostatic, astringent, nutritional, sunscreen, neuroprotective, anti-inflammatory, antiproliferative and anticancer properties.	[208,209,210,211,212,213,214,215,216,217,218,219,220,221]

## Data Availability

No new data were created or analyzed in this study. Data sharing is not applicable to this article.
